# Extramuscular Recording of Spontaneous EMG Activity and Transcranial Electrical Elicited Motor Potentials in Horses: Characteristics of Different Subcutaneous and Surface Electrode Types and Practical Guidelines

**DOI:** 10.3389/fnins.2020.00652

**Published:** 2020-07-17

**Authors:** Sanne Lotte Journée, Henricus Louis Journée, Stephen Michael Reed, Hanneke Irene Berends, Cornelis Marinus de Bruijn, Cathérine John Ghislaine Delesalle

**Affiliations:** ^1^Equine Diagnostics, Wyns, Netherlands; ^2^Research Group of Comparative Physiology, Department of Virology, Parasitology and Immunology, Faculty of Veterinary Medicine, Ghent University, Merelbeke, Belgium; ^3^Department of Neurosurgery, University Medical Center Groningen, University of Groningen, Groningen, Netherlands; ^4^Department of Orthopedics, Amsterdam University Medical Center, Amsterdam, Netherlands; ^5^Rood & Riddle Equine Hospital, Lexington, KY, United States; ^6^M.H. Gluck Equine Research Center, Department of Veterinary Science, University of Kentucky, Lexington, KY, United States; ^7^Wolvega Equine Clinic, Oldeholtpade, Netherlands

**Keywords:** transcranial stimulation, equine neurology, electromyography, subcutaneous electrodes, surface electrodes

## Abstract

**Introduction:**

Adhesive surface electrodes are worthwhile to explore in detail as alternative to subcutaneous needle electrodes to assess myogenic evoked potentials (MEP) in human and horses. Extramuscular characteristics of both electrode types and different brands are compared in simultaneous recordings by also considering electrode impedances and background noise under not mechanically secured (not taped) and taped conditions.

**Methods:**

In five ataxic and one non-ataxic horses, transcranial electrical MEPs, myographic activity, and noise were simultaneously recorded from subcutaneous needle (three brands) together with pre-gelled surface electrodes (five brands) on four extremities. In three horses, the impedances of four adjacent-placed surface-electrode pairs of different brands were measured and compared. The similarity between needle and surface EMGs was assessed by cross-correlation functions, pairwise comparison of motor latency times (MLT), and amplitudes. The influence of electrode noise and impedance on the signal quality was assessed by a failure rate (FR) function. Geometric means and impedance ranges under not taped and taped conditions were derived for each brand.

**Results:**

High coherencies between EMGs of needle-surface pairs degraded to 0.7 at moderate and disappeared at strong noise. MLTs showed sub-millisecond simultaneous differences while sequential variations were several milliseconds. Subcutaneous MEP amplitudes were somewhat lower than epidermal. The impedances of subcutaneous needle electrodes were below 900 Ω and FR = 0. For four brands, the FR for surface electrodes was between 0 and 80% and declined to below 25% after taping. A remaining brand (27G DSN2260 Medtronic) revealed impedances over 100 kΩ and FR = 100% under not taped and taped conditions.

**Conclusion:**

Subcutaneous needle and surface electrodes yield highly coherent EMGs and TES–MEP signals. When taped and allowing sufficient settling time, adhesive surface-electrode signals may approach the signal quality of subcutaneous needle electrodes but still depend on unpredictable conditions of the skin. The study provides a new valuable practical guidance for selection of extramuscular EMG electrodes. This study on horses shares common principles for the choice of adhesive surface or sc needle electrodes in human applications such as in intraoperative neurophysiological monitoring of motor functions of the brain and spinal cord.

## Highlights

- Surface and sc needle recordings in horses represent extramuscular highly coherent EMG activity.
- Adhesive gel electrodes approach the quality of sc needle electrodes for any EMG activity under specific conditions.
- The signal quality depends on the adhesive electrode type.
- A few brands of adhesive gel electrodes can replace subcutaneous needle electrodes for TC-MEP recording when taped.
- Failure rates of surface-electrode MEPs are higher when compared to sc needle electrodes.

## Introduction

Transcranial stimulation (TS) has become a standard technique for assessment of the motor function of the spinal cord. Using transcranial magnetic stimulation (TMS), this technique was introduced in the equine community by Mayhew and Washbourne (1996) and evolved as a diagnostic tool in horses (Nollet et al., 2002, 2003a,b, 2004). Recently, transcranial electric stimulation (TES) was introduced as an alternative method (Journée et al., 2014, 2015, 2018). Both TS techniques are applied under sedation and are well tolerated and painless in horses. The elicited MEPs can be recorded either intramuscular (im) or extramuscular (em). Insulated needle electrodes with uncoated tips belong to the im class (Skinner et al., 2008). These sense the electrical activity from just a few muscle fibers and reflect characteristics of the peripheral motor neuron and are therefore appropriate for diagnostic assessment of the lower motor neuron function (Wijnberg et al., 2003; Wijnberg and Franssen, 2016). The wave shapes of im recorded transcranial MEPs (TC-MEP) show typical polyphasic patterns with relative high amplitudes, which greatly differs from recordings from extramuscular electrode types (Skinner et al., 2008). MEPs can also be recorded extramuscular either at the surface of the skin by adhesive surface electrodes or subcutaneously (sc) by uncoated needle electrodes. Extramuscular MEPs typically reflect electrical activity of many motor units representing a large population of motor neurons whereas characteristics of single motor neurons are filtered out. Therefore, em MEP recordings are appropriate for assessing the upper motor neuron function since these sense the integral activity of many axons of spinal motor tracts and interconnected neurons and are insensitive for the activity of individual lower motor neurons (Skinner et al., 2008; Gonzalez et al., 2018).

As noticed, wave shapes and amplitudes of em and im MEPs are fundamentally different: in contrast to em recordings, im MEPs are highly polyphasic due to the dominant influence of only a few motor neurons. However, motor latency times (MLT) appear interchangeable between em and im electrodes (Skinner et al., 2008; Rijckaert et al., 2018). Although em EMG recordings can be obtained with both surface or sc needle electrodes and thus both are applied in many human and animal studies, to our knowledge, no profound comparison between the two electrode types has been performed up until now. Only certain viewpoints on the subject have been addressed (Ashram and Yingling, 2008; Crum and Strommen, 2008).

In horses, TC-MEPs have been reported for both intramuscular and extramuscular electrodes. First transcranial elicited MEPs (TC-MEPs) in horses were recorded extramuscular by alligator clips placed on skin folds (Mayhew and Washbourne, 1996) and later on by sc needle electrodes (Journée et al., 2014, 2015, 2018) and im needle electrodes (Nollet et al., 2002, 2003a,b, 2004). More recently in horses, adhesive surface electrodes seem an attractive practical alternative for sc needle or alligator clip electrodes since these simply can be placed right away on the dense haired skin without any further preparation. These electrode types have successfully been applied in equine ECG recordings (Verheyen et al., 2010). This is surprising because the skin is covered with sebum, epidermal tissue, residual sweat, and a dense hair layer. These compounds have highly isolating characteristics, which are known to degrade the signal quality of surface electrodes. This is a well-known problem in human patients, which can be counteracted by skin preparation such as abrasion and prior cleansing with ethanol (Hermens et al., 2000; Huigen et al., 2002) or more successfully by using abrasive conductive paste (Piervirgili et al., 2014). Sometimes, these actions are not sufficient to overcome the disturbing electrode noise, which can be a problem when signal amplitudes are expected to be very small like during intraoperative monitoring of patients with expected weak muscle MEPs like in paresis cases. This can occur in patients suffering from, for example, cerebral aneurysms, spinal cord tumors, and neuromuscular scoliosis. To overcome this doubt, sc electrodes are often an *a priori* choice in those circumstances (Deletis and Sala, 2008; Neuloh and Schramm, 2009; Szelényi et al., 2010; Takeda et al., 2018). Inspired by the successful application of adhesive surface electrodes in ECGs, Rijckaert et al. (2018) reported the use of adhesive surface electrodes for recording of TMS-MEPs in horses. These were well tolerated. However, only MLTs of im and em recorded MEPs from TMS could be compared, due to the study setup. This is because other MEP-characterizing parameters also strongly depend on whether the EMG recordings are from intramuscular or extramuscular origin. Therefore, a genuine conclusion on relevant characteristics of surface electrodes is precluded in that study. This problem is avoided when MEPs from surface electrodes are compared with MEPs of other extramuscular electrode types such as sc needle electrodes. Also in the aforementioned study (Rijckaert et al., 2018), MLTs were recorded sequentially in time and not pairwise and are therefore dominated by random physiological test-to-test variations and errors from magnetic coil repositioning (Kaneko et al., 1996, 1997). These sequential variations can be ruled out by pairwise comparison of simultaneously recorded EMGs from sc needle and surface electrodes with a sub-millisecond precision. Finally, as mentioned by Rijckaert et al. (2018), it is known that surface electrodes can have high noise levels, which may therefore reduce the quality of the obtained signal. Especially in horses suffering from either spinal cord or upper motoneuron pathology and in which manifestation of weak MEPs is to be expected, occurrence of high noise levels might impede proper diagnosis. It is well known that background noise of sc electrodes is significantly lower when compared to the background noise of adhesive surface electrodes and that high electrode impedance most often is associated with increased electrode background noise (Geddes, 1972). Most of the noise is likely of electrochemical origin at the electrode contact interface and depends on the composition of the electrode gel (Piervirgili et al., 2014). In human for example, impedances of adhesive surface AG/AgCl electrodes can be 1–2 magnitudes higher than that reported for sc needle electrodes (Grimnes, 1983; Huigen et al., 2002; Journée et al., 2004). High electrode impedances also increase the sensitivity for power line noise (Piervirgili et al., 2014; Merletti et al., 2016). Extra fastening of the electrodes by circumferential applied tape, further called “taping,” might reduce electrode impedance and thus reduce the background noise when compared to absent mechanical fixation (not taped condition). The low impedances of sc needle electrodes are associated with typical low background noise levels in clinical practice (Crum and Strommen, 2008).

The expected attractive features of adhesive surface electrodes in practice are still worthwhile to further explore. This was reason to set up a novel study design (1) to compare simultaneously obtained recordings of spontaneous EMG activity and TES-induced MEPs of a series of sc needle electrode and adhesive surface electrodes of different brands, (2) to assess impedance values and associated background noise of these electrodes under both not taped and taped conditions, and (3) to provide practical recommendations with respect to the tested electrode types and brands.

## Materials and Methods

### Materials

Six warmblood horses, consisting of two stallions, one gelding, and three mares, aged 10.07 ± 5.81 years (mean ± SD), were included in the study. The height at withers was 160.9 ± 10.2 cm (mean ± SD). Five horses showed clinical signs of muscle weakness and ataxia and were suspected of suffering from spinal or intracranial injury. These horses were subjected to a standard protocol for multipulse TES–MEP assessment in the Equine Clinic in Wolvega, the Netherlands. A 6th horse without clinical signs of weakness or ataxia was not subjected to the neurophysiological assessment and solely included for performance of impedance measurements of surface-electrode types.

### Methods

Horses were subjected to a standardized neurological exam and were prepared as previously described (Journée et al., 2018). The neurophysiological tests were performed under sedation by intravenous administration of detomidine (Detosedan, AST Farma B.V., Oudewater, Netherlands) and butorphanol (Butomidor, AST Farma B.V., Oudewater, Netherlands) (both 1.5–2.0 μg/kg body weight in total).

#### Stimulation

Two stimulation needle electrodes (L 35 mm, Ø 0.45 mm, type RMN35/0.45 Electrocap BV, Nieuwkoop, Netherlands) were inserted sc parallel to each other and caudo-rostrally on the forehead. The stimulation needle electrodes were separated 5 cm from each other, with their middle points 2.5 cm bilateral from the central location Cz on the forehead. The horses were discharged after completion of the procedure and a final clinical examination.

Transcranial electric stimulation was performed according to a standardized diagnostic protocol using biphasic multipulse trains of constant voltage generated by an intraoperative neurophysiological monitoring system (Neuro-Guard JS Center, Bedum, Netherlands). Multipulse TES was performed with three biphasic pulses per train (ppt), pulse width of (pw) 0.1 ms/phase, and interpulse interval (ipi) of 1.3 ms. The stimulation voltage was stepwise increased according to a protocol as previously defined (Journée et al., 2018). At each voltage step, TS was performed 2–4 times. The diagnostic MEP parameters were retrieved at respectively 10, 20, and 30 V above the motor threshold (MT). No further TES stimulations were added to the clinical diagnostic protocol for the current study. Each TES session was extended with free-running EMG recordings and impedance measurements of the electrodes to explore differences between surface-electrode types.

#### Data Acquisition

**FIGURE 1:**
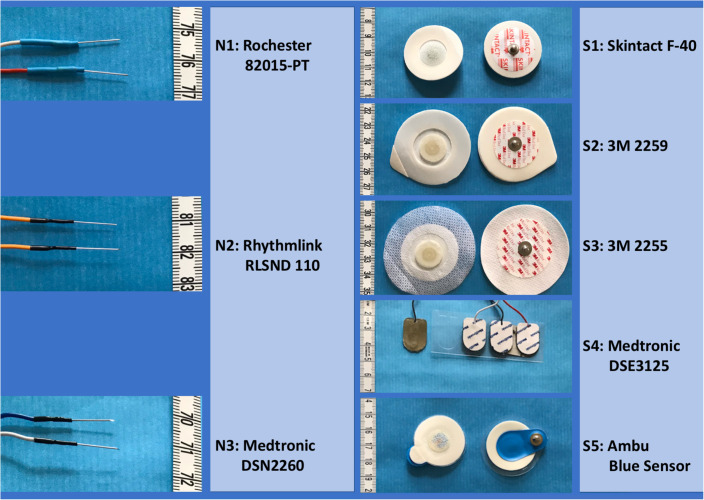
Photographic pictures of studied surface-electrode types. Detailed specifications including the dimensions of the needle electrodes are described in the methods section.

Spontaneous EMG activity and TES-induced MEPs were recorded bilaterally on all four limbs from sc needle electrode pairs which were pair-by-pair placed, interspaced about 3–4 mm: (N1) Rochester 82015-PT L 12 mm 27GA Rochester Lutz, FL, United States, (N2) RLSND 110 L 13 mm, Ø 0.4 mm Rhythmlink, Columbia, SC, United States, (N3) Medtronic dual electrode 13 mm 27G DSN2260, Medtronic Xomed, Inc., Jacksonville, FL, United States (Figure 1). One pair consisted of one type, while complimentary pairs consisted of different types. To enable the pairwise EMG and MEP recordings for this study, the sc needle electrodes were overlaid by adhesive surface electrodes of type (S1) Skintact^TM^ FS50 wet gel foam (Innsbruck, Austria) while keeping needle insertion entries a few millimeters outside the conducting pad area. The skin was left uncleansed and unabraded with the covering hair unclipped prior to placement of the surface electrodes. The sc needle and surface-electrode combinations were placed over the musculus extensor carpi radialis (ECR) (10 and 20 cm above the os carpi accessorium) and the musculus tibialis cranialis (TC) (10 and 20 cm above the medial malleolus). Sc needle electrode pairs and corresponding surface-electrode pairs were connected to the differential inputs of the physiological amplifiers of the measuring system. This allowed for simultaneous recording of four-channel needle and surface-electrode pairs, totaling eight channels. A ground needle electrode was placed sc in the neck at the right side of the horse.

The input impedance of the physiological amplifiers is 20 MΩ at 50 Hz. The common mode rejection rate is 80 dB. The analog-to-digital conversion was performed in combination with an eight-channel multiplexer using a resolution of 1 μV. The sample frequency per channel was 4.3 kHz.

#### EMG Recordings

For the study, each session was extended with free-running EMG recordings and impedance measurements of the electrodes to explore differences between surface-electrode types. A bandpass filter was used with a high-pass filter of 50 Hz and a low-pass filter of 2500 Hz (3-dB cutoff level). No further analog or digital filtering techniques were applied. Since in the first three horses it became obvious that the S1-type surface electrode showed relative high background noise, the idea was raised that other surface-electrode types might perform better. Therefore, it was decided to also have an orienting look at impedances of an additional series of different adhesive surface-electrode types in the 4th and 5th ataxic horses. Due to the limited available time, the number of additional surface-electrode types to be tested was restricted to a maximum of 2, leaving limited possibilities to perform repeated measurements. This was the reason to include a 6th horse in which solely surface electrodes were applied for impedance measurements without performing TES measurements. This allowed to also repeat the impedance measurements over a time frame of about 10–15 minutes, which agrees with the necessary adaptation times reported for gelled electrodes (Huigen et al., 2002; Roy et al., 2007) under not taped and taped conditions. This allowed in the 6th horse for execution of four impedance measurements per electrode type and impedance measurements of a total of 16 surface electrodes (4 impedances/type). In the 4th, 5th, and 6th horses, additional surface-electrode brands were attached to the skin near the Skintact electrodes. The additional tested surface-electrode brands were as follows: (S2) 3M^TM^ Red Dot^TM^ 2259-50 solid gel soft cloth; (S3) 3M^TM^ Red Dot^TM^ Foam Ag/AgCl Monitoring Electrode, 3M Deutschland GMBH Neuss, Germany; (S4) Medtronic Dual Electrode DSE3125 adhesive hydrogel on silver/silver chloride Medtronic-Xomed, Inc., Jacksonville, FL, United States; and (S5) Ambu^TM^ Blue Sensor P-00-S/50 hydrogel, Ambu S/A Ballerup, Denmark. The available extra time for the sessions of the first five horses was limited to 30 min. This set a limit to the number of free-running background signal measurements of the extra surface electrodes leaving only the possibility of impedance measurements. A survey of the five surface-electrode types is given in Figure 1.

In all neurophysiological assessed horses, TES–MEPs and time series with spontaneous EMG activity and/or background noise, which still may contain low-amplitude EMG activity, were recorded in four signal pairs from all extremities using different sc needle and Skintact adhesive surface electrodes. Continuous time series of all needle and surface-electrode types were recorded not taped and sealed by tape wrapped around the limb. TES–MEP series were obtained with tape-sealed electrodes on all occasions.

#### Impedance Measurements

After calibration with a 2-kΩ test resistor, impedance measurements were performed of all examined electrode pairs under not taped and taped conditions. The impedance resulted from rectification and RC low-pass filtering of the amplified AC voltage as measured over electrode pairs when applying a small 2.5-kHz AC constant current. The device was part of the equipment applied for intraoperative neurophysiological monitoring and intended to check the electrode impedances in patients. The frequency of the AC current was determined by an oscilloscope at 2.5 kHz. The time interval between placement of the electrodes and impedance measurements was at least 5 minutes for adaptation of the electrode–tissue interface. Impedance measurements and background noise recordings were repeated after 10–15 min to check for reproducibility.

### Data Processing

Considered TES–MEP parameters were wave shape, MLT, MEP amplitude, and cross-correlation values of MEPs. In addition, impedance, background noise, and failure rate (FR) were assessed for the different electrode types.

#### Motor Latency Times

The MLTs were defined as “the time lag between the onsets of stimulation and MEP response” when these were unambiguously distinguishable from baseline noise level.

#### TES–MEP, EMG, and Background Noise Amplitudes

The amplitudes of TES–MEPs were retrieved within the transcranial time window (TCW) between cessation of the stimulation artifact and onset of extracranially elicited late MEP responses, which are reflexes typical for horses, to preclude their interfering effects (Journée et al., 2018). Amplitude parameters were measured as peak–peak differences (A_PP_) and root-mean-square (RMS) values (A_RMS_).

The made assumptions of nearby identical myographic signals of sc needle and surface electrodes apply to all kinds of EMG activity as were verified for TC-MEPs, reflexes, and background EMG activity. When cross-correlations and RMS amplitudes of sweeps were compared, time windows were selected over signal parts of interest.

Background noise amplitudes were determined on silent parts of the signal recordings, where no EMG activity was observed. When necessary, time windows were adapted to exclude interference by transient EMG activity. RMS amplitudes of selected signal intervals were graphically visualized in a scatter plot visualizing the relation between background noise and electrode impedance.

### Statistical Analyses

Statistical analysis was performed with SPSS^TM^ software, version 20.0.0, IBM^TM^.

#### Comparison of EMG Wave Shapes of sc Needle and Surface Myograms and Reproducibility

The cross-correlation function R_xy_(τ) quantifies the cross-correlation between sample time series x(t) and y(t) according to the equation:

(1) $$R_{\mathrm{xy}}\,\left(\tau \right)=\frac{\sum_{t=W_{\mathrm{start}}}^{W_{\mathrm{end}}-1}x\,\left(t\right)\cdot y\,\left(t+\tau \right)}{\sqrt{\sum_{i=W_{\mathrm{start}}}^{W_{\mathrm{end}}-1}x^{2}\,\left(i\right)}\cdot \sqrt{\sum_{j=W_{\mathrm{start}}+\tau }^{W_{\mathrm{end}}+\tau -1}y^{2}\,\left(j\right)}}$$

where x(t) and y(t) are the time functions of myograms from the sc needle and surface electrodes or from two subsequent myograms of one channel, t is the discrete value of time, and τ is the time shift between the two time functions, and W_start_ agrees with the sample number at the start and W_end_ with the sample number at the end of the time window W in which the cross correlation is computed. τ is computed between −14 and 14 ms in time steps of 1/f_s_, where f_s_ is the sample frequency. W_start_ was chosen at 19 ms to exclude influences of TES artifacts of multipulse TES and the length of W 150 ms. These choices are applied in all autocorrelation and cross-correlation calculations. The maximum of R_xy_(τ) was used to quantify the correlation between sc needle and surface-electrode signal pairs or between subsequent recorded myograms. R_xy_(0) is similar to Pearson’s correlation for τ = 0. When the channel numbers are different, τ is corrected for the multiplex delay bias in the 8-channel sample cycle. The algorithm is programmed in Borland C++, version 3.1. The autocorrelation and cross-correlation functions R_xx_(τ), R_yy_(τ), and R_xy_(τ) and RMS amplitudes together with the specifications of the stimulus parameters and time window W of each sample series were transferred to SPSS databases for subsequent statistical processing. The full-width half (FWHM) of the autocorrelation and cross-correlation functions served as a measure to indicate the width of the signal waves of the MEPs, spontaneous EMG activity, and background noise and used when reviewing the relation with the reviewed time functions.

#### Comparison of TES–MEP Parameters of Subcutaneous Needle and Surface Electrodes

A descriptive analysis of TES–MEP parameters was performed on MLTs, amplitude ratios, and cross-correlation factors between signal pairs. For each case and muscle group (m), the following values were computed: (a) mean MLT for needle electrodes, mMLT_n,m_ with standard deviation *SD*_n,m_ (*N* = 6) and (b) mean differences of MLTs between surface (s) and needle (n) electrode pairs mMLT_s–n,m_ with standard deviation *SD*_s–n,m_ (*N* = 6). The averages were obtained from repeated stimulations (2 per voltage) at 10, 20, and 30 V above MT. The standard deviations *SD*_n,m_ depend also on variations of the MLT in time where MLTs differences are computed after averaging. This sensitivity for time-varying influences is excluded in the standard deviation *SD*_s–n,m_ of MLT differences of electrode pairs, which are computed before averaging. The normality of the relevant differences was graphically assessed using qq plots (MLT and MEP amplitudes in the linear and background noise amplitudes and impedances in the logarithmic domain). MLTs and their intra-individual and interindividual differences were case-wise compared in paired-samples Student *t*-tests under the null hypotheses of means being zero.

#### Failure Rate, Impedance, and Background Noise Under Not Taped and Taped Conditions

The impedances and background electrode noise and their statistical tests resulting in differences and 95% confidence intervals were processed in the logarithmic domain before back conversion in the linear domain. When converted back to the linear domain, differences become ratios. In the linear domain, G_mean_ becomes the geometric mean of RMS amplitude values. GM_U/T_ denotes the improvement of the quality of the electrodes after taping defined as the ratio of not taped G_mean_/taped G_mean_, where the indices _U_ refer to not taped and _T_ to taped (sealed) conditions. Impedance range ratios are defined as the quotient of the upper and lower limits. Background amplitude-impedance pairs of electrodes under taped and not taped conditions were plotted bi-logarithmic from which a FR function, expressing the fraction of rejections of baseline noise amplitudes exceeding an *A*_RMS_ = 12 μV RMS (*A*_PP_ ≅ 50 μV) level, was computed as running average with a window containing 8 subsequent impedances. The averages were fitted by a local regression curve using bi-weight 50% smoothing parameters in a locally weighted scatterplot smoothing (LOESS) procedure (SAS Institute Inc, 1999).

In all statistical comparisons, a significance level of *p* = 5% was applied.

## Results

### Similarity Between Myographic Signals of Subcutaneous Needle and Surface Electrodes

**FIGURE 2:**
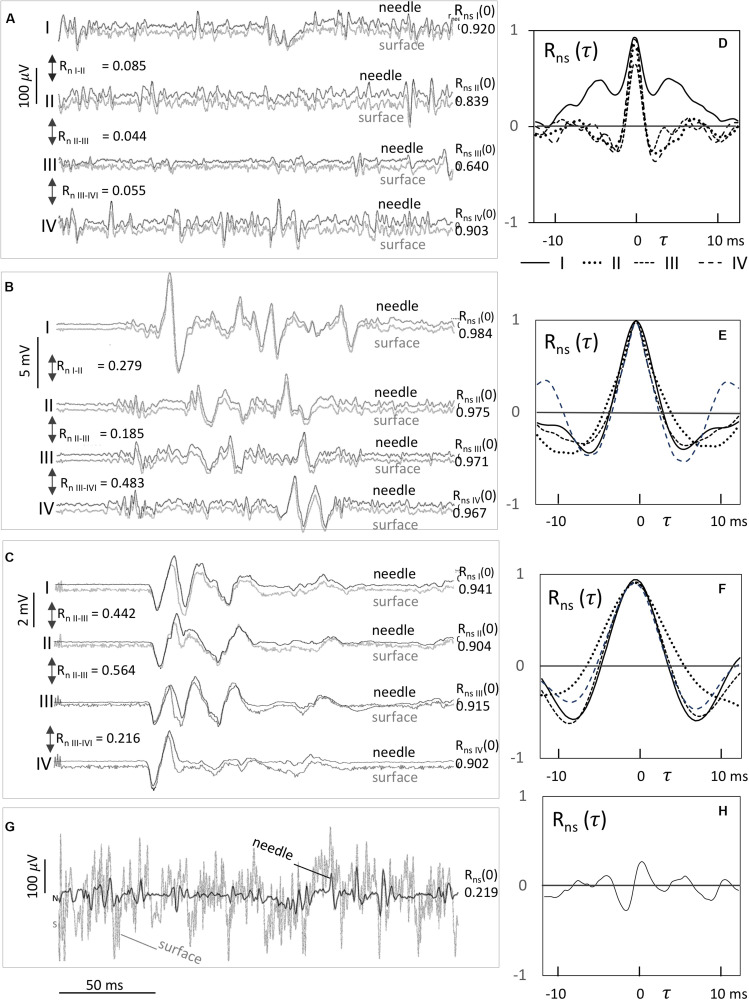
Examples of paired recordings from sc needle and surface electrodes **(A–G)** with corresponding cross-correlation functions *R*_ns_(τ) **(D,E,F,H)**. Each signal graph **(A–C)** shows the signal pairs labeled as I, II, III, and IV of the needle electrodes in black and surface electrodes in gray shades of four subsequent recordings. The correlation values of *R*_ns_(τ) at τ = 0 of the signal pairs I–IV are given at the right side. The correlation values *R*_n,I–II_, *R*_n,II–III_, and *R*_n,III–IV_ at τ = 0 between two subsequent needle electrode signals are mentioned in between the signal pairs. **(A)** EMG background activity, **(B)** dispersed polyphasic TES–MEPs due to a compromised spinal cord in a severely ataxic horse, **(C)** TES–MEPs with prolonged MLTs of a horse with mild ataxia, and **(G)** background EMG activity with a high background noise level of a surface electrode recording at a high impedance (29 kΩ) resulting in a non-significant small cross-correlation value of *R*_NS_ (0) = 0.219.

Figures 2A–C show the expected high coherent resemblance of each of the four subsequent recordings (sweeps) of myographic signal pairs of the surface and sc needle electrode recordings for either stationary continuous EMG activity (Figure 2A) or TES–MEPs (Figures 2B,C). TES–MEPs are shown in a severely ataxic horse (Figure 2B) and in a mild ataxic horse (Figure 2C). The coherent appearance of the sc and surface signal pairs is objectified by the high correlation seen at the maxima of the cross-correlation functions R_sn_(τ) in respectively Figures 2D–F. Each function belongs to one sweep. After correction for the time bias between multiplexed samples, all cross-correlation functions depict sharp delineated maxima at τ = 0. The high cross-correlation values provided in Figures 2D–F agree with the highly coherent courses of all four subsequent signal pairs depicted in Figures 2A–C.

Most correlation values are only partly affected by moderate background noise levels, unless when strong surface-electrode noise fully masks the visibility of EMG activity as seen in Figure 2G. Figure 2G shows the devastating effects of strong surface-electrode noise on continuous EMG activity, resulting in non-significant low cross-correlation values around zero as seen in graph 2 h. This belongs to one exception of the right ECR of case 2 (Table 1). Moderate noise levels partly impair the correlation as shown in case 2 (Table 1) where correlations are reduced to 0.699–0.796 and 0.847–0.899 for the right and left TC muscles. These are still significant for *p* < 0.01.

**TABLE 1:** 95% confidence intervals of cross-correlations at τ = 0 values between surface and needle electrodes *R*_ns_(0) and amplitude ratios *A*_s/n_ of the RMS amplitudes of the surface and needle electrodes in five horses (cases).

**Muscle group**	**Case 1**		**Case 2**		**Case 3**		**Case 4**		**Case 5**	
	
	**95% confidence intervals**									
	***R*_ns_(0)**	***A*_s/n_**	***R*_ns_(0)**	***A*_s/n_**	***R*_ns_(0)**	***A*_s/n_**	***R*_ns_(0)**	***A*_s/n_**	***R*_ns_(0)**	***A*_s/n_**
ECR L R	0.937	0.919	0.963	0.932	0.950	0.919	0.980	0.961	0.961	0.941
	0.967	0.999	0.967	1.04	0.975	0.999	0.985	0.977	0.983	0.962
	0.959	0.952	**−0.022**	**2.37**	0.965	0.944	0.990	0.999	0.840	0.953
	0.967	1.00	**−0.181**	**4.15**	0.976	0.993	0.993	1.03	0.901	0.978
TC L R	0.955	0.624	0.699	0.835	0.917	0.611	0.991	0.996	0.906	0.903
	0.973	0.831	0.796	0.987	0.966	0.813	0.993	1.02	0.946	0.931
	0.979	0.927	0.847	0.773	0.882	0.927	0.903	0.881	0.975	0.979
	0.989	1.028	0.899	0.941	0.932	1.03	0.945	0.923	0.988	0.994

*The figures for each side are obtained from 2 subsequent TES-MEP recordings at 20 V above MT. The bold figures result from strong interfering noise in the surface EMG observed along with high surface-electrode impedance supporting the observation of the high amplitude ratios and not significant low correlations.*

Table 1 provides a statistical overview of cross-correlation values R_ns_(0) and amplitude ratio’s A_s/n_ of all four muscle groups in five cases. A ratio of 1 denotes equal A_RMS_ amplitudes in both signals of a pair. The overview of cross-correlation values of surface and sc needle MEP-signal pairs of all but one of 20 muscle groups depicted in Table 1 shows them to be highly significant since most values are close to 1.

A_RMS_ of surface electrodes are somewhat lower than those of sc needle electrodes shown by A_S/N_ values ranging between 0.8 and 1.0 (18/20 muscle groups) and in a left TC muscle group (case 2) between 0.7 and 0.8. The high A_S/N_ range between 2.4 and 4.2 recorded at the right ECL muscle group of case 2 is ascribed to the high surface-electrode noise level.

The width of the bell-shaped R_ns_(τ) curves is related to the widths of the waves in the continuous EMG and MEP recordings. Smaller full-width-half-maximum (FWHM) values are noticed in the continuous EMG in a neighborhood of 1 ms, whereas FWHM values of MEPs are about 3–4 ms. Cross-correlation functions between subsequent TC-MEPs vary and show random variations of τ of about 2 ms. These variations appear synchronous to MLT fluctuations.

The mean of the paired differences mMLT_s–n_ (Table 2) is in 85% of 20 muscle groups within −0.2 and 0.2 ms. *SD*_s–n_s are below 0.3 ms in 55% and below 0.6 ms in 90% of the muscle groups. This is much lower than the SDs in subsequent MLT measurements of below 3.0 ms in 70% and below 6 ms in 85% of cases. One exception with *SD*_s–n_ = 2.0 ms (left TC, case 2) is found where MLTs of small strong varying prolonged polyphasic wave patterns become inaccurate due to high surface-electrode noise. The variations of *SD*_n_ of sequential MLTs are about one magnitude higher than those of SD_s–n_s of paired differences: *SD*_n_ values for thoracic limb muscles are below 3.1 and for pelvic limb muscles below 9.5 ms. The 9.5 ms is an outlying value in a case with prolonged MLTs.

**TABLE 2:** Survey of mean MLTs of needle electrodes (mean_tn_) and mean of paired MLT differences (mean_ts–tn_) between surface and needle electrodes with respective standard deviations *SD*_n_ and *SD*_s–n_ in five horses.

**Muscle group**	**Case 1**				**Case 2**				**Case 3**				**Case 4**				**Case 5**			
	**Mean tn ts-tn ms**		**SD tn tn-ts ms**		**Mean tn tn-ts ms**		**SD tn tn-ts ms**		**Mean tn tn-ts ms**		**SD tn tn-ts ms**		**Mean tn tn-ts ms**		**SD tn tn-ts ms**		**Mean tn tn-ts ms**		**SD tn tn-ts ms**	
ECR L R	23.1	0.05	2.79	0.23	24.6	−0.05	2.16	0.23	26.6	−0.02	2.85	0.27	21.8	0.14	0.44	0.29	20.9	0.11	1.31	0.10
	23.0	0.16	3.14	0.37	24.8	−0.27	1.41	0.34	27.8	0.00	2.92	0.35	22.3	0.09	1.40	0.23	20.3	0.08	0.513	0.57
TC L R	46.3	0.11	9.47	0.93	49.6	−0.90	2.40	1.97*	60.2	−0.05	4.74	0.32	44.0	−0.17	6.29	0.28	34.9	0.03	1.46	0.22
	44.3	0.05	8.72	0.56	51.4	−0.64	1.37	0.53	56.2	0.36*	5.95	0.59*	40.2	−0.17	2.33	0.28	35.2	−0.02	1.19	0.40

* Worsened latency time readability caused by high interference noise in the surface EMG. The number of cases for each mean is 6. The ratios between the ECR and TC muscle groups 11.9x (left), 8.6 (right), 9.9 (left), and 15.7 (right) show about one magnitude differences between *SD*_*tn*_ and *SD*_*ts–tn*_.

### Comparison of MLT

Table 2 shows means of MLTs of all muscle groups and cases together with means of paired MLT differences between surface and sc needle electrodes with the respective standard deviations *SD*_N_ and *SD*_S–N_. Test-to-test variations are incorporated in *SD*_N_ and excluded in *SD*_S–N_ within-test MLT pairs.

### Relation Between Electrode Noise and Impedances

**FIGURE 3:**
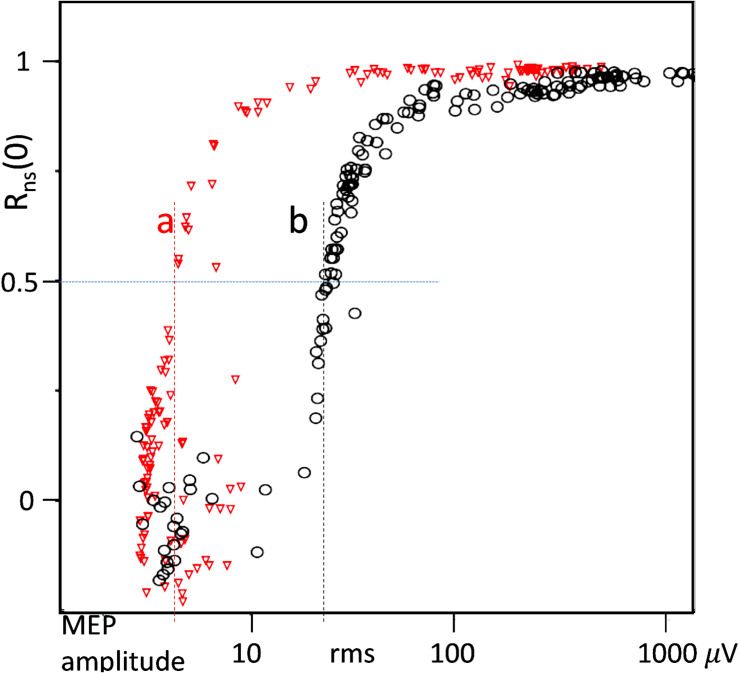
Cross correlation *R*_ns_(0) between needle versus surface-electrode signals n(t) and s(t) of MEPs obtained from muscle MEP versus TES-voltage curves of the standard measuring procedure (Journée et al., 2015, 2018). **(a)** Moderate surface-electrode noise level: background noise needle 2.5 μV; surface: 18 μV RMS and **(b)** high surface-electrode noise level showing its masking effects on lower MEP amplitudes. Background-noise needle: 3.5 μV; surface: 108 μV RMS. The noise levels are read at correlations of 0.5 and depicted by the vertical dashed lines.

Figure 3 depicts how the cross-correlation R_ns_(0) between the surface and sc needle electrode MEPs is influenced by two different electrode noise levels of respectively A_RMS_ 18 μV (a) and *A*_RMS_ = 108 μV (b).

**FIGURE 4:**
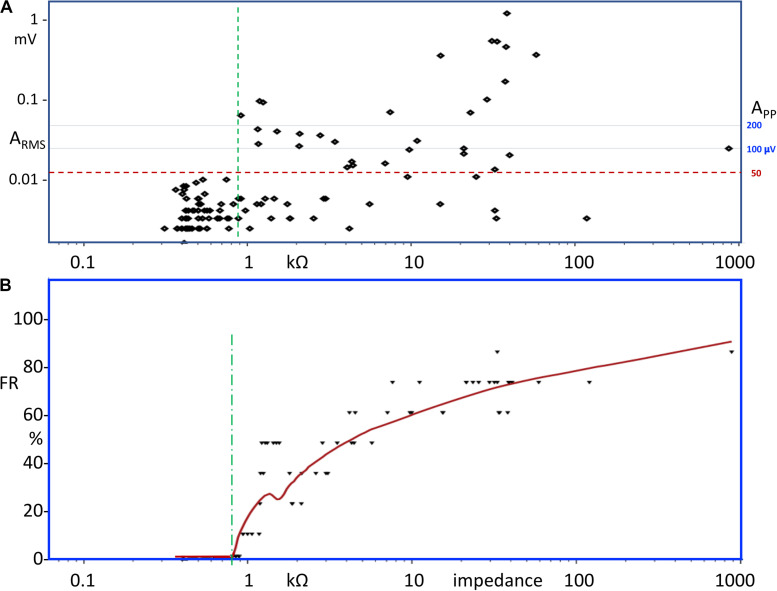
**(A)** Scatter plot of RMS MEP amplitudes as a function of the electrode impedance (*N* = 114 points). **(B)** Running average of the failure rate (FR) over a frame width of eight subsequent impedance values. The FR values are plotted at the end of the frames. FR is computed as the percentage of MEP amplitudes within the frame above *A*_RMS_ = 12 μV, which agrees with about *A*_PP_ = 50 μV (horizontal dashed line). The vertical dashed lines denote the impedance borders below which in panel a no background noise amplitudes exceed *A*_RMS_ = 12 μV and in **(B)** FR = 0. **(B)** LOESS local regression curve of the FR as function of electrode impedance. A vertical dashed line indicates the impedance value at which the FR becomes greater than zero.

The relation between electrode background noise amplitude and impedance is depicted in the double logarithmic scatter plot in Figure 4A for sc needle and surface electrodes in not taped and taped conditions.

### Evolution of the Surface-Electrode Impedance Over Time

The impedances of a total of 16 surface electrodes (4 impedances/type) showed over the course of 15 min highly significant (*p* < 0.001) decreases in time with mean rates of: not taped 95.4% and taped 97.3% decrease. This decrease was not seen in the S4-type electrodes. The impedance reduction over time in our study agrees with the 40% reduction reported by Laferriere et al. (2011) for dry surface electrodes. In that study, the greatest impedance reductions are reported during the first 10 min in an exponential decaying fashion. This may continue over 30 min thereafter (Laferriere et al., 2011). The values found in the current study match with those reported for pre-gelled ECG electrodes by Grimnes (1983). In one horse, we continued with additional impedance measurements for about 1 h after the initial impedance series. As expected, S1 electrodes showed further impedance reductions ranging from −9% to −67% resulting in impedance values ranging from 313 to 595 Ω. Comparable impedance reductions were also reported for pre-gelled ECG electrodes (Grimnes, 1983).

### Impedance Ranges of sc Needle and Surface Electrodes Under Not Taped and Taped Conditions

Impedance ranges of all electrode types, impedance range percentage of impedances surpassing 10 kΩ, and FRs are provided in Table 3 for all tested sc needle (N1–3) and surface electrode (S1–5) types under either not taped or taped conditions.

**TABLE 3:** Survey of electrode impedance range percentages of impedances (test frequency 2.5 kHz) surpassing 10 kΩ, and failure rates (FR) of needle (N1–N3) and surface electrode (S1–S5) types under not taped and taped conditions.

**Electrode type**	**Condition**	**Impedance range Ω**			**GM_U/T_**	**p**	**% >10 kΩ**	**FR at *A*_RMS_ >12 μV**
		**Lower range limit**	**G_mean_**	**Upper range limit**				
N1	Not taped	423	613	915	1.20x	0.1	0%	0%
	Taped	398	510	679			0%	0%
N2	Not taped	420	429	502	1.04x	0.48	0%	0%
	Taped	375	409	509			0%	0%
N3	Not taped	412	431	566	0.88x	0.25	0%	0%
	Taped	402	487	548			0%	0%
Overall needle		375	505	915			0%	0%
S1	Not taped	876	3.6k	14.8k	3.65x	0.007**	24%	65%
	Taped	312	986	4.4k			7%	30%
S2	Not taped	1.95k	45.9k	>100k	29.9x	0.001**	83%	72%
	Taped	467	968	6.58k^1^			17%	27%
S3	Not taped	1.35k	2.70k	38.5k^1^	5.93x	0.000**	46%	58%
	Taped	338	456	854			23%	0%
S4	Not taped	>100k	>100k	>100k	0.94x	0.76	100%	100%
	Taped	92k	>100k	>100k			100%	100%
S5	Not taped	1.98k	5.72k	12k	5.13x	0.000**	50%	69%
	Taped	545	1.11k	2.28k			0%	21%

Excluded outliers: ^1^1 case > 100 kΩ. ** Significant for *P* < 0.01. G_*mean*_: geometric mean. GM_*U/T*_ ratio: not taped G_*mean*_/taped G_*mean*_.

### Background Noise

The background noise of surface electrodes usually is inversely related to the impedance. However, the relationship between both parameters shows a large variability (Huigen et al., 2002; Laferriere et al., 2011). This is also observed in the current study in which the relation between all A_RMS_ amplitude–impedance pairs is shown in the scatter plot of Figure 4A. There is an increasing scattering of a cloud of points to over 2 decades width. This is also reported by Huigen et al. (2002). Up until 900 Ω (vertical dashed line), the amplitudes of none of the points exceed *A*_RMS_ = 12 μV, which agrees with *A*_PP_ ≅ 50 μV (this value is often used as discrimination criterion for TES thresholds; Szelényi et al., 2007; Aydinlar et al., 2018). The conversion factor from A_RMS_ to A_PP_ is *F*_P–RMS_ = 4.1.

Figure 4B shows the running average of the FRs at discrimination level *A*_PP_ > 50 μV of the total number of data pairs. The wide scatter above 900 Ω comprises a mix of acceptances and rejections of all electrodes at discrimination level *A*_PP_ = 50 μV. The LOESS regression curve through the points of the running average FR function visualized in Figure 4B emerges at 810 Ω (vertical dashed line) by an initial steep increase that gradually loses steepness at increasing impedances. FR levels of 20% are already reached at 1 kΩ, 50% at between 4 and 5 kΩ, and 80% at over 100 kΩ. In Table 3, the FR values are computed as ratios at a discrimination level at *A*_PP_ = 50 μV. The ratios represent the fraction of rejections within the impedance ranges of all electrode types and taping conditions. For all tested sc needle electrodes, the FR = 0 and corresponding impedances were well below 900 Ω. Non-significant differences at large overlapping ranges are found between needle electrode types and also between all tested hydrogel electrode types. However, there is one important exception for the Medtronic surface electrodes (type S3) which show all high impedances in a range well above 100 kΩ. The GM_U/T_ ratios depict the improvement of the signal quality of surface electrodes by taping. Overall impedance ranges of sc needle electrodes are much smaller than those of surface electrodes. This is clearly reflected in Table 3 where range ratios are between 1.3 and 1.9x. Taping does not change the impedances of needle electrodes significantly but remains useful to prevent their dislodging.

We experienced different qualities of adhesive properties where the fixating properties of the S3 electrode was the best and worst for the S4 electrode which often detached spontaneously.

## Discussion

### Similarity of Myographic Recordings Between sc Needle and Surface Electrodes

The current study provides an objective comparison of different electrode types and brands and for extramuscular measurements and offers physicians a new valuable practical guidance for selection of extramuscular EMG electrodes. Besides the diagnostic application in horses, the equivalence between EMG and TES–MEPs of sc needle and surface electrodes is also still an underexplored subject in human intraoperative neurophysiological monitoring (Deletis and Sala, 2008; MacDonald et al., 2013).

It was postulated that extramuscular recorded EMGs in horses from surface and sc needle electrodes share the same wave morphology and amplitudes and are about equal with A_RMS,__s_ somewhat smaller than A_RMS,n_. Similar findings are described for ECG recordings by surface and sc needle electrodes (Bellardine Black et al., 2010). This implies that the signals of identically placed em electrodes remain coherent, regardless of whether it concerns stationary spontaneous EMG activity, or transcranial or extracranial elicited motor responses. Underlying pathology plays no role. The aforementioned similarity of wave morphology is not present when comparing between im and em needle electrode recordings. Intramuscular EMGs differ markedly from extramuscular recordings due to the presence of polyphasic waves (Giroux and Lamontagne, 1990; Skinner et al., 2008; Gonzalez et al., 2018). Similarity of waveforms is essential for specific comparison of electrode qualities and to judge whether they are interchangeable and free from bias from differences between intramuscular and extramuscular characteristics. This was a methodological limitation in the study of Rijckaert et al. (2018) in which surface-electrode MEPs from TMS were compared with im MEPs. Only comparison of MLTs of surface and im MEPs was justified in that study setup, as also has been shown in several human studies (Ertekin et al., 1998; Verin et al., 2002; Brostrom et al., 2003).

As expected, the results of our study show convincing coherent similarity between extramuscular EMGs of surface and sc needle electrodes. This pertains to continuous EMG and all TES–MEP-characterizing parameters as wave morphology, amplitudes, and MLTs. The equivalence of MEP waveforms is visible in signal pairs of continuous background EMG activity with relative low amplitudes (Figure 2A), in the dispersed polyphasic transcranial and reflex MEPs in an ataxic horse (Figure 2B), and in pathological delayed MEPs of relative larger amplitudes in a horse with mild ataxia (Figure 2C).

The high-correlation factors and amplitude ratios near *A*_S/N_ = 1, depicted in Table 1 of surface-needle MEP pairs, allow to conclude that both em electrode types deliver (1) equal wave forms of MEPs and (2) about equal MEP amplitudes, with A_RMS_ of surface electrodes somewhat lower than sc needle electrodes. The outlier with the high A_S/N_ range of the right ECL muscle group of case 2 is ascribed to the high surface electrode noise; (3) sharp delineated maximum cross-correlation factors R_NS_(τ) are all at τ = 0. This implies that surface electrodes are well aligned over needle electrodes. When somewhat displaced from each other, this could theoretically cause a time shift between the two coherent signals when the motor endplate zone is not enclosed by the electrode pairs. When the velocity of traveling action potentials along muscle fibers is for example 4 m/s, the passage time difference by 4 mm displaced electrodes would be 1 ms. Accordingly, R_NS_(τ) is maximal at τ_max_ = 1 ms. For relative narrow cross-correlation functions, like in Figure 2D, the maximum cross-correlation R_NS_(τ_max_) would greatly be underestimated by R_NS_(0). In horses, no data are available of muscle fiber conduction velocities and geometry of motor endplate zones in muscles. This was the specific reason to include the possibility to detect non-zero τ_max_ values in the methods of this study.

For the MLT, the sequential statistics are comparable with those reported in the study of Rijckaert et al. (2018) who noticed similar prolonged MLT outliers in the pelvic limb muscle groups in assumed healthy horses.

Cross-correlation functions depict within which time-range signals remain coherent. When these time ranges are clearly contained within the sweep lengths, the sweep-to-sweep correlations of stationary noise signals, like from continuous EMG activity, are uncorrelated. This implies that the wave morphology in each sweep is different. This also applies for the continuous EMG in Figure 2A where the correlation values are very low between −0.055 and 0.085.

Basically, cross-correlations are connected to the reproducibility of the wave shapes of signals. High cross-correlations indicate a high similarity between the morphology of the signals. A cross-correlation of 1 implies a strict linear relation between signals. This means that their shapes are exact copies of each other, while their amplitudes may differ. The cross-correlation can be considered as a measure of reproducibility of the wave morphology. Low correlations indicate a poor while higher values reflect a better reproducibility of the wave shapes. The wave shapes are different when the correlation is not statistical significant.

The correlation between successive MEPs in Figure 2B is also low and varies between −0.2 and 0.5. This agrees with the poor reproducibility of highly variable complex polyphasic and delayed weak MEP waves in a marked ataxic horse. Higher correlation values between 0.2 and 0.6 in Figure 2C reflect better reproducible morphology of polyphasic MEPs in a horse with mild ataxia.

Differences in wave morphology of subsequent MEPs as expressed by the correlation between subsequent sweeps make comparison of wave parameters as MLT and MEP morphology less reliable, as has been performed in the study of Rijckaert et al. (2018). This was also mentioned by these authors as a study limitation. Pairwise comparisons within each MEP measurement eliminates the susceptibility to differences between subsequent MEPs. In the current study, this was prevented by the simultaneous pairwise comparison of electrode recordings by overlaying the sc needle electrodes with the adhesive electrodes. This approach offers a sub-millisecond accuracy as illustrated by the *SD*_s–n_ values.

### Effects of Surface-Electrode Noise

When MEP amplitudes are sufficiently high as in healthy horses, surface electrodes appear as an appealing alternative for sc needle electrodes (Rijckaert et al., 2018). However, when the signal-to-noise ratio (SNR) is poor, surface-electrode noise may become a problem. This can be expected in pathological conditions where MEPs are small and polyphasic. Surface electrodes have usually higher electrode impedances, which is usually accompanied by increased noise levels (Grimnes, 1983). Electrode impedance and background noise also depend on the electrode types (adhesive gel or dry surface electrodes), the electrode contact surface size, and conditions at the skin, such as the presence of perspiration (Grimnes, 1983; Godin et al., 1991; Huigen et al., 2002; Roy et al., 2007; Piervirgili et al., 2014; Merletti et al., 2016). Figure 2G shows the devastating effects of pronounced surface-electrode noise on continuous EMG recordings blinding its visibility, which complies with the absence of significant cross-correlation (Figure 2H). The influence of surface-electrode noise becomes more evident at lower EMG amplitudes. This is demonstrated in the continuous EMG recordings in Figure 2A where the relatively high EMG amplitudes of the 1st and 4th surface-needle signal pairs show correlation values above 0.9, while these values drop to respectively 0.84 and 0.64 at lower EMG amplitudes in the 2nd and 3rd pairs. The surface-electrode noise in Figure 2B is higher than in Figure 1A, but the dominating influence of large MEP amplitudes ensures high correlation values well above 0.96. The about three times smaller MEP wave amplitudes in Figure 2C show somewhat lower correlations due to the relative larger influence of surface-electrode noise. This agrees with the observations in Figure 3 depicting the influence of moderate (*A*_RMS_ = 18 μV) and strong (*A*_RMS_ = 108 μV) electrode noise on cross-correlation *R*_ns_(0). The A_RMS_ threshold at *R*_ns_(0) = 0.5 is increased from 3.0 μV to one magnitude higher at 28 μV. This illustrates the vulnerability of weak MEPs for even moderate background noise from surface electrodes.

The study shows that taping is very effective to improve the signal quality of surface electrodes: (1) the impedances of a few surface electrodes even underpass those of sc needle electrodes. This may be ascribed to a good adaptation of the electrolyte interface between electrode and skin in combination with the larger contact surface of the electrodes. Larger contact surfaces imply lower impedances (Geddes, 1972; Merletti et al., 2016). The contact surface of a disk electrode, Ø 10 mm, is about fivefold that of the contact surface of a needle electrode of length 12 mm, Ø 0.4 mm. Between electrode impedances of 300 and 500 Ω, the relation between impedance and electrode contact surface transits from an uncorrelated independent to a linear relationship, in which the segmental impedance between the electrodes at 2.5 kHz becomes overruled by the local electrode impedance (Journée et al., 2004; Berends and Journée, 2018). Only the latter is proportionally related with the electrode contact surface (Journée et al., 2004; Berends and Journée, 2018). The finding of a further impedance decrease after 1 h in one horse confirms that the signal quality of surface electrodes further improves after a longer settling time. However, this would imply the need for longer session times for diagnostic tests. (2) Taping reduces impedance range ratios from 31–47x to 9.1–12x, but these still remain markedly larger than the ratio of 2.4x for all needle electrodes, leaving a high-end tail being susceptible for failures. (3) After taping, the FR drops from 59–74% to 18–24%. However, with exception of S3 electrode types, a FR of zero like in sc needle electrodes is still not obtained. Even when increasing the discrimination level of *A*_PP_ = 50 μV to 100 μV or to 200 μV, FRs further reduce by 33 and 49% but still not become zero. The decrease in impedance seen in taped surface electrodes is consistent with the described influence of pressure on surface electrodes (Laferriere et al., 2011). The decrease in the impedance depends on the type of electrode (wet Ag/AgCl, conductive textile or dry) and shows type-dependent, sometimes large, gradual irreversible lasting decreases in time, which partly are ascribed to an increased effective electrode area (Taji et al., 2018).

This study supports the conclusion that in healthy horses where MEP amplitudes are large compared to electrode noise, adhesive surface electrodes can replace sc needle electrodes, but only when the electrodes are being taped (Rijckaert et al., 2018). It is not clear whether in that study the electrodes were taped. Only when not taped, the high FRs that were seen in the current study contradict the conclusion of the aforementioned study. However, even under taped conditions, except for S3 electrode types, the FR of surface electrodes could not be reduced to zero like for sc needle electrodes. In one of 20 muscle groups, a high electrode impedance and high noise level precluded accurate determination of MLTs with surface electrodes. This most probably is caused by the fact that impedances depend on many conditions that are not controlled when prior skin preparation such as cleansing, abrasion, and hair clipping are not performed. The electrolytic interface between surface electrodes and skin depends on unknown residues from sweating, which after adaptation to the electrode gel may contribute to a good conductance. On the other hand, relative important isolating properties of hair and dead epidermal debris from the skin can decrease the conductivity. We did not test the effects of hair clipping, skin cleansing, and abrading because these are considered as cumbersome and extra time consuming in practice while cosmetic marks will temporary be left behind. These aspects do not outweigh the simplicity of application and superior signal quality of sc needle electrodes. This was also the reason not to further explore the improvement of the signal quality of surface electrodes after longer adaptation times in spite of the observed further improvement of impedances in one horse after 1 h.

The orienting look at different types of surface and sc needle electrodes permits to conclude that all types, except S4, can be used for extramuscular EMG recording after additional securing by tape. However, the zero FRs of sc needle electrodes are still not obtained for types S1, S2, S4, and S5. All impedance measurements with S4 surface electrodes remained well above 100 kΩ. With one exception of 100 kΩ, most authors accept a maximum skin impedance below 10 kΩ in practice (Hermens et al., 2000). A limitation of that review is that frequencies that apply to the impedances are not specified. Since the S4 electrode often detached spontaneously and showed unacceptable high impedances, we deem this electrode type inappropriate for MEP recording in horses. The adhesive properties of the S3 surface electrodes and, when taped, their low impedance range and zero FR quality make them appealing alternatives for sc needle electrodes. However, this should be studied in a higher number of horses for statistical support.

Moreover, the possibility of circumferential taping applies only to muscle groups in extremities. Reduction in FRs of surface electrodes by taping elsewhere on the body is precluded. This leaves sc needle electrodes on those locations as the only reliable choice.

## Conclusion

In horses, and likely also in intraoperative neurophysiological applications, sc needle and gelled surface electrodes reflect extramuscular EMGs as highly coherent EMG and MEP signals whereas adhesive surface electrodes only approach the signal quality of sc needle electrodes when taped and allowing enough settling time. However, the signal quality of surface electrodes remains vulnerable to unpredictable conditions of the skin. Differences between surface-electrode types are apparent and need to be elaborated in a larger study population and also confirmed in human patients. The current study provides a new valuable guidance for physicians for selection of extramuscular EMG electrodes when performing neuro-electrical diagnostic tests or for intraoperative use. The study shares common principles for the choice of adhesive surface or sc needle electrodes in human applications such as in intraoperative neurophysiological monitoring of motor functions of the brain and spinal cord.

## Study Limitations

- Small number of included horses.
- When excluding the S4 surface electrode, the different electrode types are only studied in three horses and give not yet sufficient insight in horse-to-horse variations and statistical support for a generalized opinion on differences between four adhesive gel surface electrodes.
- Surface electrode characteristics may approach the subcutaneous needle electrode quality when allowing long electrode settling times like 1 h and preparation with skin cleansing, abrading, and hair clipping before starting MEP measurements. This is not examined.

## Data Availability Statement

The datasets generated for this study are available on request to the corresponding author.

## Ethics Statement

The horses that were included in the study were client-owned horses that were presented to the Clinic for diagnostic work-up for complaints of spinal ataxia. With that respect horses were subjected to the standard diagnostic work-up protocol for spinal ataxia of which multipulse TES-MEP assessment is a standard part. No extra, nor additional interventions aside from the veterinary necessary work-up approach were applied in these horses. Only a set of extra (non- invasive) adhesive electrodes was placed on the legs. Impedance measurements of the electrodes were performed in conjunction with the multipulse TES-MEP assessment work-up. The 6th horse is the privately owned horse of the first author. This horse was only provided with a set of adhesive surface electrodes placed on the skin of her legs (non-invasive). No multipulse TES-MEP assessment was performed. The owner(s) informed consent for participation in this study, either written or oral was given.

## Author Contributions

SJ and HJ: equal contribution to this article in design, acquisition, analysis, interpretation, and writing. HB and SR: critical revision important for the intellectual content based on their professional background. CB: provision of experimental facilities, giving practical advices, and revision of the manuscript. CD: involvement in interpretation, writing, and revision.

## Conflict of Interest

The authors declare that the research was conducted in the absence of any commercial or financial relationships that could be construed as a potential conflict of interest.

## Abbreviations

τ: time shift parameter in autocorrelation and cross-correlation functions
A_PP_: peak-to-peak amplitude
A_RMS_: RMS amplitude
A_S/N_: RMS amplitude ratio between surface and needle electrode pairs
dB: decibel
ECR: musculus extensor carpi radialis
em: extramuscular
EMG: electromyogram
F_P–RMS_: conversion factor from A_RMS_ to A_PP__;_
FR: failure rate
FWHM: full-width-half-maximum
G_mean_: geometric mean of amplitude
GM_U/T_: ratio of G_mean_ values under taped and not taped conditions
Hz: hertz
im: intramuscular
ipi: interpulse interval
_m_: index muscle group m
MEP: (muscular) motor evoked potential
MLT: motor latency time
mMLT_n_: mean MLT needle electrode MEP
mMLT_s–n_: mean of MLT needle-surface-electrode pair differences
MT: motor threshold
_n_: index needle electrode
ppt: pulses per train
pw: pulse width
RMS: root-mean-square
R_xx_(τ): autocorrelation function of signal x(t)
R_xy_(τ): cross-correlation function between signals x(t) and y(t)
R_yy_(t): autocorrelation function of signal y(t)
_s_: index surface electrode
sc: subcutaneous
SD: standard deviation
SNR: signal to noise ratio
TC: musculus tibialis cranialis
TC-MEP: transcranial MEP
TCW: transcranial time window
TES: transcranial electric stimulation
TMS: transcranial magnetic stimulation
TS: transcranial stimulation
W: time window.
